# Metagenomic Analysis to Assess the Impact of Plant Growth-Promoting Rhizobacteria on Peanut (*Arachis hypogaea* L.) Crop Production and Soil Enzymes and Microbial Diversity

**DOI:** 10.1021/acs.jafc.4c05687

**Published:** 2024-09-26

**Authors:** Ezequiel D. Bigatton, Romina A. Verdenelli, Ricardo J. Haro, Ibrahim Ayoub, Florencia M. Barbero, Maria Paula Martín, Lucas E. Dubini, Jesús V. Jorrín Novo, Enrique I. Lucini, María Ángeles Castillejo

**Affiliations:** † Facultad de Ciencias Agropecuarias, Microbiología Agrícola, Universidad Nacional de Córdoba, Ingeniero Agrónomo Félix Aldo Marrone 746, Córdoba X5000, Argentina; ‡ Consejo Nacional de Investigaciones Científicas y Técnicas (CONICET), Av. Ciudad de Valparaíso S/N, Córdoba X5016, Argentina; § Departamento de Bioquímica y Biología Molecular-ETSIAM, AGR-164 Bioquímica, Proteómica y Biología de Sistemas Vegetal y Agroforestal, Universidad de Córdoba, Autovía N−IV Km 396, Campus Rabanales, Córdoba, Andalucía 14071, Spain; ∥ Instituto Multidisciplinario de Biología Vegetal (IMBIV-CONICET-UNC), Instituto de Ciencia y Tecnología de los Alimentos (FCEFyN-UNC), Av. Vélez Sarsfield 1666, Córdoba X5016, Argentina; ⊥ Estación Experimental Agropecuaria INTA Manfredi, Instituto Nacional de Tecnología Agropecuaria (INTA), Ruta Nacional N°9 Km 636, Manfredi, Córdoba X5988, Argentina

**Keywords:** Bacillus, Pseudomonas, crop yield, soil metagenome, soil enzymes, FDA, DHA

## Abstract

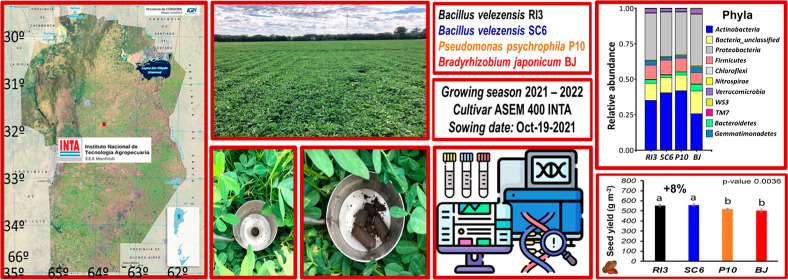

Peanut production could be increased through plant growth-promoting rhizobacteria (PGPR). In this regard, the present field research aimed at elucidating the impact of PGPR on peanut yield, soil enzyme activity, microbial diversity, and structure. Three PGPR strains (*Bacillus velezensis*, RI3; *Bacillus velezensis*, SC6; *Pseudomonas psychrophila*, P10) were evaluated, along with *Bradyrhizobium japonicum* (BJ), taken as a control. PGPR increased seed yield by 8%, improving the radiation use efficiency (4–14%). PGPR modified soil enzymes (fluorescein diacetate activity by 17% and dehydrogenase activity by 28%) and microbial abundance (12%). However, PGPR did not significantly alter microbial diversity; nonetheless, it modified the relative abundance of key phyla (Actinobacteria > Proteobacteria > Firmicutes) and genera (*Bacillus* > *Arthrobacter* > *Pseudomonas*). PGPRs modified the relative abundance of genes associated with N-fixation and nitrification while increasing genes related to N-assimilation and N-availability. PGPR improved agronomic traits without altering rhizosphere diversity.

## 1 Introduction

Global food production is constantly increasing, albeit slower than the world’s population.^1^ Enhancing the quantity and quality of agricultural outputs is pivotal in ensuring food security. Peanuts and other legumes (e.g., soybeans, dry beans, dry peas, chickpeas, etc.) emerge prominently within the spectrum of foods contributing to balanced nutrition. The peanut cultivation area covers over 27 million hectares worldwide.^1^ Argentina is the first Latin American and seventh-largest peanut producer (ca. 3% global production) and the first-world peanut exporter (990,000 Mg year^–1^).^1^ Córdoba province concentrates 250,000–270,000 ha (75% of the total sowing area) with yields ranging from 2.7 to 3.45 Mg ha^–1^. In these temperate regions, peanut production depends on a frost-free period from early October to early April (20.4 °C and 670 mm, 90 years of weather historical average from Oct to Apr.) (ca. 160–180 days).^2,3^ These environmental characteristics (e.g., lower grown temperatures than other peanut-producing regions) allow Argentine peanut grains to accumulate interesting proportions of sugars and calcium instead of fatty acids,^4^ which contributes to producing a sweet and crunchy grain. In addition, Argentina’s climate provides an appropriate environment for accumulating tocopherols (vitamin E), which are antioxidants that allow a longer grain life, maintaining quality. These chemical traits of Argentinean peanuts are positioned as among the most desired by consumers.^4^

Considering the necessity of increasing food production, peanut farmers should increase and sustain the crop yield while reducing the environmental impact. Plant growth-promoting rhizobacteria (PGPR) are microorganisms that inhabit the rhizosphere and rhizoplane, interacting with the crops to promote growth and yield. The PGPR promotes crop growth through direct (e.g., phytohormones production, nutrient solubilization, nitrogen fixation, siderophores production, etc.) and indirect (e.g., hydrolytic enzymes, antibiotics, induction of systemic resistance, production of siderophores, VOCs, etc.) mechanisms.^5^ Legume crops such as peanuts, *Bradyrhizobium*, *Azospirillum*, *Bacillus*, and *Pseudomonas* are the most common PGPR genera.^6−10^ These microorganisms are typically formulated as inoculants and applied to seeds before sowing to establish associations from seed germination.^11^ PGPR application positively modifies the soil microbial population and rhizospheric dynamic, aiming to enhance crop growth.^12^ Several works reported that the PGPR application on peanut crops increased yields between 15% and 35%. These improvements primarily stem from changes in yield components, growth rates, and resource use efficiency resulting from PGPR’s direct or indirect mechanisms of action.^10^

Crop yield improvement throughout the PGPR implies elucidating the PGPR mechanisms involved in this plant–microbe relationship. In the soil, native microorganisms interact with the plant rhizosphere and the PGPR inputs.^13^ Metagenomics is a promising tool for comprehending plant–microbiome interactions to attain sustainable crops and peanut production.^14^ These -omics analyses integrated into the crop production process allow us to understand the functional and structural changes in the rhizosphere linked to the host behavior. Soil microbiomes mainly change due to the plant growth phenological stages.^15^ Also, PGPR applications affected the soil microbiome.^15^ PGPR effects on crops depend on the performance and persistence of the selected strains to spread along the rhizosphere and compete with native microorganisms.^16^ Several reports showed that PGPR on crops (e.g., maize, rice, wheat, potato) changed the abundance of the soil’s bacterial communities.^17^ PGPR shapes the rhizospheric soil and induces changes in the soil microbiome diversity, population, and structure. Legume crops (e.g., soybeans, peas, and peanuts, among others) demonstrated an increase in the presence of microorganisms involved in the N transformation (i.e., N-fixation, ammonia-oxidizing, denitrification, organic nitrogen transformation, etc.) among other mechanisms.^18,19^

PGPR application on peanuts commonly modifies Proteobacteria, Actinobacteria, Acidobacteria, Firmicutes, and Chloroflexi as the major phyla that affect crop growth.^20,21^ Most peanuts-PGPR microbiome studies are approached at the plant scale (pots) or plant level.^20−23^ In this study, Bacillus and Pseudomonas genera were applied to peanut crops to evaluate the effects of these PGPRs on the rhizobacterial community associated with the crop under field-level conditions. This study aimed to determine the effects of PGPR on peanut yield generation, soil biological properties, and microbiome structure through a metagenomic approach under field conditions compared to standard inoculation with *Bradyrhizobium japonicum*. Our study made certain assumptions: first, PGPR enhances crop yield by increasing seed yield (SY) components (e.g., seed number (SN), weight, etc.); second, PGPR modifies the diversity, structure, and composition of soil microbiome, which promotes plant growth.

## 2 Materials and Methods

### 2.1 Experimental Site

**Figure 1:**
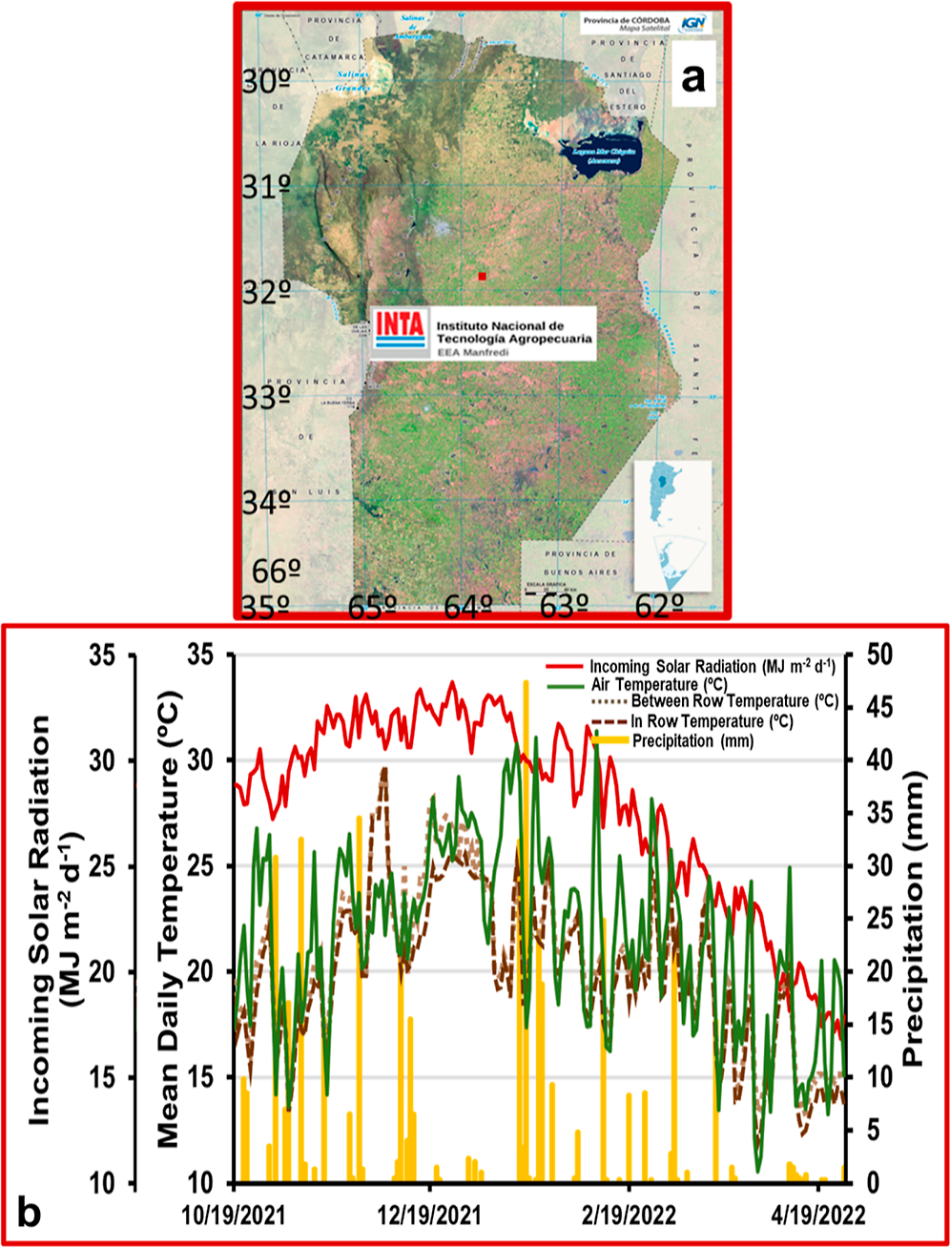
Experimental site and weather conditions. (a) INTA Research Station in Manfredi, Córdoba, Argentina (31° 49′S, 63° 46′W) (dark red square). (b) Daily incoming solar radiation, mean air temperature, between-row soil temperature, in-row soil temperature, and precipitation recorded during the crop cycle from sowing (Oct-19-2021) to harvest (Apr-27-2022). In the set, figure (b) indicates the reference for each variable.

A field experiment was conducted during the 2021–2022 peanut crop season (Oct-19-2021 to Apr-27-2022) at the Instituto Nacional de Tecnología Agropecuaria research station (INTA) in Manfredi, Córdoba, Argentina (31° 49′S, 63° 46′W) (Figure 1). The soil type is Silty Loam Typic Haplustoll, and its chemical properties are summarized in Table 1. This area is characterized by a semiarid monsoon climate with 765 mm of annual precipitation and 16.9 °C mean yearly temperature.^3^ The incident of global solar radiation, and solar radiation, as reported in the NASA POWER database, was converted into photosynthetically active radiation by multiplying by 0.45^24^ (Figure 1). Daily mean air temperature and precipitation were obtained from a weather station of INTA^3^ close to the experiment (Figure 1). The soil temperature was measured daily throughout the dataloggers (Cavadevises) strategically soil positioned 5 cm depth (depth of pod setting) of the surface in the crop row and between the crop rows (Figure 1).

**Table 1:** Soil Chemical Properties (0–20 cm Depth) of INTA Research Station from Manfredi, Córdoba, Argentina (31° 49′S, 63° 46′W)

soil chemical properties (0–20 cm depth)	
pH	6.60
Ec (dS m^–1^)^a^	0.50
SOM (%)	2.40
TOC (%)	1.41
C/N	11.00
EP (ppm)	25.00
TN (%)	0.13
N–NO_3_^–^ (ppm)	13.80
S–SO_4_^–^ (ppm)	1.22
Zn (ppm)	2.06

a Ec: electrical conductivity; SOM: soil organic matter; TOC: total organic carbon; C/N: carbon–nitrogen ratio; EP: extractable phosphorus; TN: otal nitrogen.

### 2.2 Crop Husbandry, PGPR Strains, and Experimental Design

A commercial Runner-type peanut cultivar, ASEM-400 INTA (hereafter named Asem 400; intermediate growth cycle between 145 and 150 days from sowing to harvest), was used. Three PGPR strains were applied: *Bacillus velezensis* RI3 strain (RI3), *Bacillus velezensis* SC6 strain (SC6), Pseudomonas psychrophila PSE10 strain (P10), and *B. japonicum* (BJ) considered one of the most common peanut inoculants.^6^ All the strains were provided by the Universidad Nacional de Córdoba, Facultad de Ciencias Agropecuarias (UNC-FCA), and Laboratorio de Microbiología Agrícola with a final concentration of 1 × 10^9^ colony forming unit (CFU) mL^–1^. These strains were previously field-tested in an ecophysiological trial by Bigatton et al.^10^ and selected for their ability to improve peanut yields significantly. Following the standard peanut sowing practices, seeds were treated with a compatible combination of thiabendazole (15 g L^–1^), fludioxonil (2.5 g L^–1^), and methalaxil-M (2 g L^–1^). Before sowing, seeds were pelleted with the selected PGPR; each strain was applied separately. To verify the targeted concentration of 1 × 10^6^ CFU seed^–1^, 10 g of the treated seeds were resuspended in peptone water. Serial dilutions were released for bacterial plate count on tryptic soy agar medium for Bacillus sp., King F medium for Pseudomonas sp., and nitrogen-free medium for Bradyrhizobium sp. Inoculation treatments were arranged in a randomized complete block design with three replicates. Plots had four 13 m long rows at 0.7 m spacing (36.4 m^2^) with a stand density of 14 plants m^–2^. During the crop cycle, plots were maintained free of weeds, pests, and diseases.

### 2.3 Crop Measurements

To analyze growth parameters, crop phenology was recorded weekly in each plot from the emergence (Ve) to the final harvest (R8).^25^ Light interception measurements and biomass sampling at each phenological stage were determined following Bigatton et al.^10^ Briefly, at final harvest, plants in 1 m^2^ were harvested from the two central rows of each plot to determine: (i) total biomass (TB; in g m^–2^); (ii) corrected TB (TBc; in g m^–2^); (iii) pod yield (PY, in g m^–2^); (iv) pod number (PN, in m^–2^); (v) SY (in g m^–2^); (vi) SN (in m^2^); (vii) seed weight (SW, in g); (viii) radiation use efficiency (RUE; g MJ^–1^); (x) fraction of seeds ≥8 mm (SF_≥8mm_). TB involved the above biomass (vegetative) plus pod biomass. TBc was calculated as vegetative biomass plus reproductive biomass multiplied by a 1.65 energy correction factor.^26^ SF ≥ 8 mm included seeds retained through sequential sieves of different mesh sizes of 7-, 8-, 9-, and 10 mm diameter round-hole, and the proportion of seeds ≥8 mm was expressed in percentage.

### 2.4 Soil Sampling

At Ve (T0; emergency) and R5 (T1; beginning of seed growth) phenological stages,^25^ composite soil samples (15 subsamples) of each plot were collected from the crop rhizosphere at 20 cm depth of the two central rows (12 samples, *n* = 12). This approach aimed to ensure that the samples adequately represented the mean plot conditions. Subsequently, the samples were refrigerated at 4 °C until further processing. Soil samples were processed within the next 24 h of collection and dried at room temperature for 24 h. Stubble and roots were separated, and 20 g of the fine fraction was collected after passing the soil through a 1 mm sieve.

### 2.5 Soil Enzyme Activity Assays

#### 2.5.1 Dehydrogenase Activity

Dehydrogenase activity (DHA) was determined according to Garcia et al.^27^ Briefly, 0.2 mL of 0.4% INTF (2-*p*-iodophenyl-3nitrophenyl-5-phenyltetrazolium chloride) in distilled water was added to 1 g of soil at 60% of field capacity and incubated at 22 °C for 20 h in darkness. The formed INTF (iodonitrotetrazolium) was extracted with 10 mL of methanol and filtered through a Whatman N°5 filter paper. The enzyme activity was estimated spectrophotometrically at 485 nm. The concentration of INTF was calculated by using a calibration curve constructed with different standards (ranging from 1 a 50 μg mL^–1^).

#### 2.5.2 Fluorescein Diacetate Hydrolytic Activity

Fluorescein diacetate activity (FDA) assay was measured following the Adam and Duncan procedures.^28^ A soil sample of 2 g was incubated at 28 °C for 30 min in 20 mL of phosphate buffer (pH 7.6) and 0.2 mL of fluorescein diacetate in acetone (1 mg mL^–1^). The reaction was stopped by adding 15 mL of chloroform–methanol 2:1 v/v, and the mixture was then centrifuged for 10–15 min at 2000 rpm. The amount of fluorescein released from the FDA was measured at 490 nm in the supernatant. The values for FDA hydrolysis were determined using a calibration curve that correlated the optical density with fluorescein concentration (ranging from 0 to 10 μg mL^–1^).

### 2.6 Phospholipid Fatty Acid Analysis

Following the method optimized by Verdenelli et al.,^29^ soil samples (8 g fresh soil) were overnight extracted with 40 mL of 1:2:0.8 (v/v/v) chloroform, methanol, and phosphate buffer (pH 7.4). Lipids were fractionated using silicic acid chromatography eluted (i.e., neutral lipids, glycolipids, and polar lipids). To determine phospholipids, fatty acid methyl esters were produced through mild-alkali methanolysis, and they were subsequently extracted using hexane and dried in N_2_. Fatty acid methyl esters were analyzed using capillary gas chromatography with flame ionization detection on a PerkinElmer Clarus 500 GC, with methyl nonadecanoate (19:0) as the internal standard bacterial acid methyl ester mix (Supelco, Supelco UK, Poole, Dorset, UK). Concentrations of PLFAs were expressed in units of nmol % g^–1^ soil. Branched fatty acids *i*15:0, *a*15:0, *i*16:0, *i*17:0, and *a*17:0 represented Gram-positive bacteria, while monoenoic and cyclopropane fatty acids 16:1ω9, 16:1ω11, *cy*17:0, 18:1ω9*c*, 18:1ω9*t*, and *cy*19:0 represented Gram-negative bacteria. Actinobacteria and fungal biomass were indicated by the fatty acid 10 methyl 18:0 and the polyenoic 18:2ω6.9, respectively. The fatty acid 16:1ω5 was an indicator of the arbuscular mycorrhizal fungi. The total microbial biomass (MB) was estimated using the total concentration of PLFAs (nmol g^–1^ soil).

### 2.7 DNA Extraction, PCR, and Amplified DNA Analysis

Total DNA was extracted from fresh soil samples (1 g each) using the Dneasy PowerSoil Kit (Qiagen). Extracted DNA was spectrophotometrically quantified (λ: 260 nm), and its purity was assessed through the 260/280 nm absorbance ratio with a NanoDrop ND-1000 spectrophotometer (NanoDrop Technologies, Wilmington, DE, USA) and gel electrophoresis. The amplification of the V3 V4 region of bacterial and archaeal 16S rRNA genes was performed using the HotStarTaq Plus Master Mix Kit, primers 341F (CCTAYGGGRBGCASCAG)–806R (GGACTACNNGGGTATCTAAT), and the following amplification program: 94 °C for 5 min, followed by 35 cycles of 94 °C for 45 s, 50 °C for 60 s, and 72 °C for 90 s. A final extension at 72 °C was used for 10 min, and the reactions were held at 4 °C. The purified products were paired-end sequenced (2 × 300) in an Illumina MiSeq Sequencing platform at Novogene Bioinformatics Technology (Co., Ltd., Beijing, China).

Sequences were processed by using the software Mothur, following the 16S microbial analysis with Mothur (extended) protocol in the web-based platform Galaxy. The SILVA SSU NR reference database (V138) from the Mothur Web site was used. A total of 1,445,401 sequences were analyzed (2,030,554 in total) after quality screening (fragments > 460bp), alignment screening (V3–V4 region), chimera checking, and taxonomy filtering (removal of unknown, eukaryotic, chloroplasts, mitochondria sequences). Thus, only bacterial and archaeal sequences were retained. Sequences were clustered into 49,269 operational taxonomic units (OTUs) at a 97% similarity cutoff. Rarefaction curves of the number of OTUs observed at different sequencing depths were obtained for each sample. The 16S rRNA gene sequences were deposited ENA/NCBI (PROJECTPRJEB74143).

### 2.8 Statistics

Crop growth, community species richness, diversity indices, phylum, genera, and metabolic analysis data were statistically analyzed using a linear mixed effects model and an Infostat statistical software for each trait. The PGPRs were included in the model as fixed effects, whereas the blocks were considered random effects as follows (eq 1)
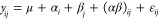
1
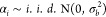

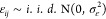
where *y*_*ij*_ is the observed value for the response variable for the *j*th PGPR (*j* = 4) combined with the *i*th block (*i* = 3); μ is the overall mean; α_*i*_ is the random effect of the *i*th block; β_*j*_ is the effects of the *j*th PGPR; (αβ)_*ij*_ represents the interaction effect between block *i* and PGPR *j*; ε_*ij*_ are the residuals error term. For each crop variable, comparative means analyses LSD-Fisher (α = 0.05) were assessed to find statistical differences among the treatments.

Also, a linear mixed effects model statistically analyzed soil enzymes and biological activity. The PGPRs and sampling time were included in the model as fixed effects, whereas the blocks were considered random effects as follows (eq 2)
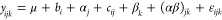
2
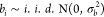

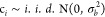

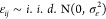
where *y*_*ijk*_ is the observed value for the response variable for *k*th PGPR (*k* = 4) combined with the *j*th T (*j* = 2) in the *i*th blocks (*i* = 3); μ is the overall mean; *b*_*i*_ is the random effect of the *i*th block; α_*j*_ is the effects of the *j*th T; *c*_*ij*_ is the random effect of the *j*th T assigned to the *i*th block which is the main plot error term; β_*k*_ is the effects of the kth PGPR; (αβ)_*jk*_ denotes the T by PGPR interaction; and ε_ijk_ are the residuals which constitute the subplot error term. For each variable, comparative means analyses LSD-Fisher (α = 0.05) were assessed to find statistical differences among the treatments.

PAST software v.4.0 was used for multivariate statistical analysis of the sequencing data. Both diversity analyses and the soil microbial communities’ functional prediction were performed using the MDP and SDP modules in the Microbiome Analyst platform, respectively. The functional role of OTUs was predicted using the Tax4Fun2 database. The Shannon index, Simpson index, and Chao1 index were calculated to assess microbial community diversity. The Shannon index was computed based on the proportion of individuals of each phylum in the community, while the Simpson index quantified the probability of two individuals belonging to the same phylum. Additionally, the Chao1 index provided an estimate of the total number of phyla in the sample, considering rare phyla. Specifically, it factored in the number of observed phyla and the frequency of phyla observed once and twice. The metabolic processes were predicted based on the COG (clusters of orthologous groups) functional categories system, which classifies proteins based on their predicted function. Data were imported using default parameters, including a low count filter (minimum count of four with 20% prevalence), a low variance filter at 10% based on interquartile range, and data scaling with total sum scaling.

The KO (KEGG Orthology)^30^ database and Tax4Fun software were used to infer the functional profiles of nitrogen cycle genes—including *nifD*, *nifH*, *ureC*, *nasA*, *nirA*, *pmoA-amoA*, *hao*, *nirK*, *norB*, and *nosZ* (Table 2). The relative abundances of each gene were calculated by dividing predicted gene copy numbers by the total normalized reads for each sample using R (version 4.3.3). Count data was normalized using the DESeq2 package and assessed for statistical significance across treatments. Fisher’s LSD test was used for multiple comparisons, and adjusted *p*-values were calculated using the Benjamini–Hochberg method to control the false discovery rate.

**Table 2:** KEGG Orthologues of N-Cycle Genes for Peanut Rhizosphere Analysis

process	reaction	KEGG orthology	gene	function
N-fixation	N_2_ → NH_3_	K02586	*nifD*	nitrogenase molybdenum–iron protein α chain
	N_2_ → NH_3_	K02588	*nifH*	nitrogenase iron protein
	N_2_ → NH_3_	K02591	*nifK*	nitrogenase molybdenum–iron protein β chain
urea hydrolysis	(NH_2_)_2_CO → NH_3_	K01428	*ureC*	urease subunit alpha
assimilatory nitrate reduction	NO_2_^–^ → NH_3_	K00366	*nirA*	ferredoxin-nitrite reductase
denitrification	NO_2_^–^ → NO	K00368	*nirK*	nitrite reductase (NO-forming)
	NO_3_^–^ → NO_2_^–^	K00370	*narG*	nitrate reductase catalytic subunit
	NO → N_2_O	K04561	*norB*	nitric oxide reductase subunit B
	N_2_O → N_2_	K00376	*nosZ*	nitrous-oxide reductase
N-assimilation	NO_3_^–^ → NO_2_–	K00372	*nasA*	assimilatory nitrate reductase catalytic subunit
	NH_3_→ Glutamine	K01915	*gln A*	glutamine synthetase
nitrification	NH_3_ → NH_2_OH	K10944	*pmoA-amoA*	methane/ammonia monooxygenase subunit A
	NH_2_OH → NO_2_^–^	K10535	*hao*	hydroxylamine dehydrogenase

## 3 Results

### 3.1 Crop Growth Analyses

**Figure 2:**
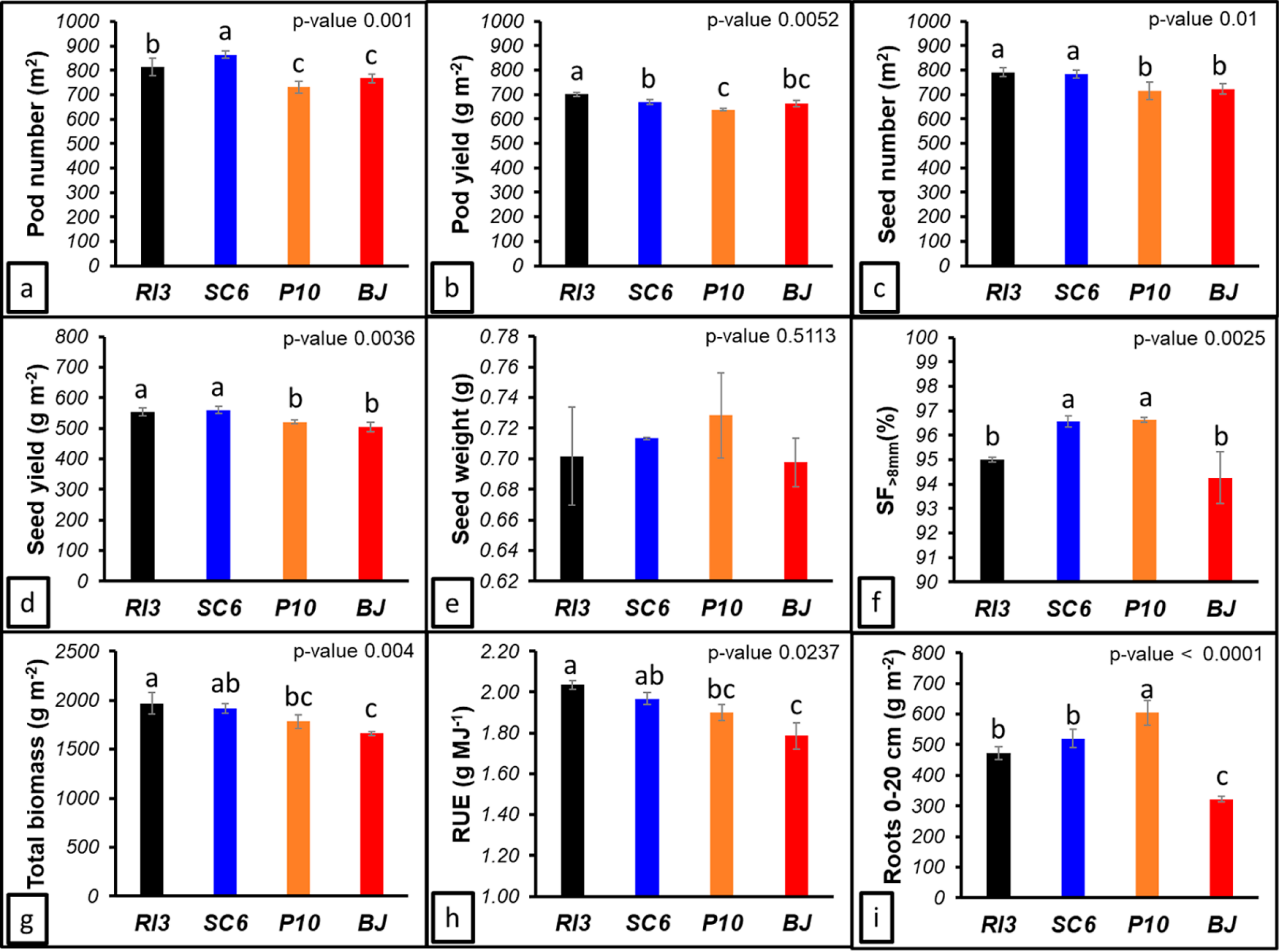
Peanut growth measurements at R8 stage. (a) PN, (b) PY, (c) SN, (d) SY, (e) SW, (f) TB (above ground), (g) seed fraction (SF > 8 mm), (h) RUE, and (i) root biomass. Legend of treatments apply in all the figures RI3, *B. velezensis*; SC6, *B. velezensis*; P10, *P. psychrophila*; BJ, *B. japonicum*. Error bars indicate standard deviations. Different letters indicate statistical differences (*p*-value < 0.05).

PN (*p* = 0.001) and PY (*p* = 0.0052) showed statistical significance. SC6 and RI3 strains achieved the highest PN (865 and 814 pods m^–2^), 13%–6% above the BJ (768 pods m^–2^), respectively (Figure 2a). The highest PY was reached by RI3 (701 g m^–2^), followed by SC6 (670 g m^–2^), BJ (663 g m^–2^), and P10 (637 g m^–2^) (Figure 2b). SC6 and RI3 exhibited the highest seed number (SN; *p* = 0.01) and seed yield (SY; *p* = 0.0036) (Figure 2c,d). RI3 achieved the highest SN with 791 seed m^–2^, 9.5% above BJ (722 seed m^–2^) (SC6 8.7% and P10-1% to BJ) (Figure 2c). The SY ranking was SC6 (559 g m^–2^) > RI3 (554 g m^–2^) > P10 (520 g m^–2^) > BJ (504 g m^–2^) (Figure 2d). SW was, on average, 0.7 g seed^–1^ (P10 > SC6 > RI3 = BJ) (Figure 2e). Regarding the seed fraction (SF > 8 mm) (*p* = 0.0025), P10 and SC6 exhibited the highest values (97.7% average) (Figure 2f).

Crop growth showed statistical differences for TB, RUE, and roots (0–20 cm) (Figure 2g–i). TB in PGPR (RI3 > SC6 > P10) was between 19% and 7% higher than BJ (Figure 2g). Considering the RUE, significant increases were achieved due to PGPR effects (*p* = 0.0237) (Figure 2h). The RUE values were 2.03 g MJ^–1^ for the RI3, 1.97 g MJ^–1^ for the SC6, 1.90 g MJ^–1^ for the P10, and 1.78 g MJ^–1^ for the BJ (Figure 2h). Roots (until 20 cm depth) exhibited the highest differences under PGPR effects (Figure 2i). P10 strain showed the best performance and promoted root growth by 88% compared to BJ (Figure 2i).

### 3.2 Soil Enzyme Assays, Lipid Analysis, and Microbial Taxonomic Structure

**Figure 3:**
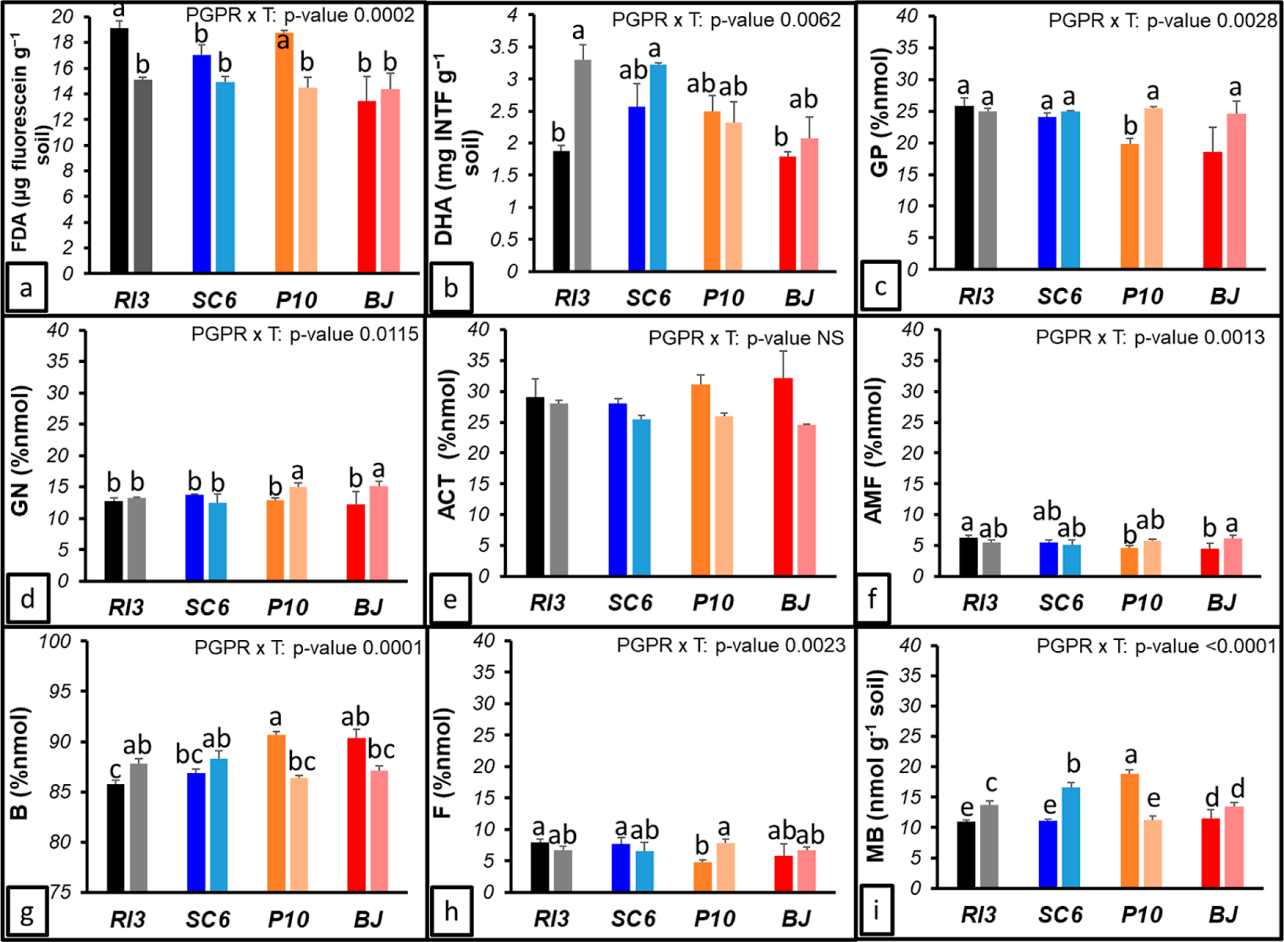
Soil enzyme activity, lipid analysis, taxonomic structure, and biomass. (a) Fluorescein diacetate analysis (FDA), (b) DHA, (c) Gram-positive microorganisms (GP), (d) Gram-negative microorganisms (GN), (e) actinomycetes (ACT), (f) arbuscular mycorrhiza fungi (AMF), (g) bacteria (B), (h) fungi (F), and (i) MB. Dark-colored bars correspond to T0, and light-colored bars to T1. T0: 15 days after sowing (crop emergency). T1: 70 days after sowing (R5 phenological stage). Legend of treatments apply in all the figures RI3, *B. velezensis*; SC6, *B. velezensis*; P10, *P. psychrophila*; BJ, *B. japonicum.* Error bars indicate standard deviations. Different letters indicate statistical differences (*p*-value < 0.05).

Soil enzyme activities were statistically enhanced under PGPR treatment (Figure 3a,b). The PGPR × sampling date (T) interaction was statistically significant for both enzymes (*p* < 0.05). In T0 (emergency) and T1 (R5; beginning of seed growth), PGPR showed a similar trend (RI3 > SC6 > P10). The abundance of active metabolic microorganisms measured by fluorescein diacetate hydrolase (FDA), exhibited a constant behavior across the experiment, with higher values at T0 compared to T1 (15.90 and 15.84 μg fluorescein g soil^–1^) (Figure 3a). RI3 in both T0 and T1 achieved the highest FDA values, 19.10 and 15.12 μg fluorescein g soil^–1^ (T0 and T1, respectively), 49% and 5% above BJ (12.78 and 14.34 μg fluorescein g soil^–1^) (Figure 3a). DHA, which showed the general soil microbial activity, attained higher values at T1 (2.79 mg INTF g soil^–1^) compared to T0 (2.18 mg INTF g soil^–1^), and PGPRs reached higher values at 29–27% compared to BJ (T0 and T1, respectively) (Figure 3b).

The PLFA analysis revealed significant differences in the soil microbial composition. The PGPR × T interaction was statistically significant (*p* < 0.05) for Gram-positive microorganisms (GP), Gram-negative microorganisms (GN), arbuscular mycorrhizal fungi (AMF), bacteria (B), fungi (F), and MB (Figure 3c–i). The abundance of GP microorganisms was 34% higher for RI3 than BJ at T0, but 8% lower at T1 (Figure 3c). For GN, P10 and BJ at T1 exhibited the highest concentration, 16–31% above T0 (Figure 3d). The abundance of AMF was 40% higher for RI3 × T0 than BJ × T0 but 13% lower at T1 (Figure 3f). B abundance showed that RI3 and SC6 at T0 were 5% lower than P10 and BJ; however, at T1, they were 2% higher (Figure 3g). The F abundance was 49% higher for RI3 and SC6 than P10 and BJ at T0; in contrast, 10% lower at T1 (Figure 3h). Considering the MB, BJ at T0 showed the highest abundance (21.46 nmol g soil^–1^), 51% above RI3 at T0, but in contrast, RI3 at T1 was 32% higher than BJ (Figure 3i).

### 3.3 Community Species Richness and Diversity Index

**Figure 4:**
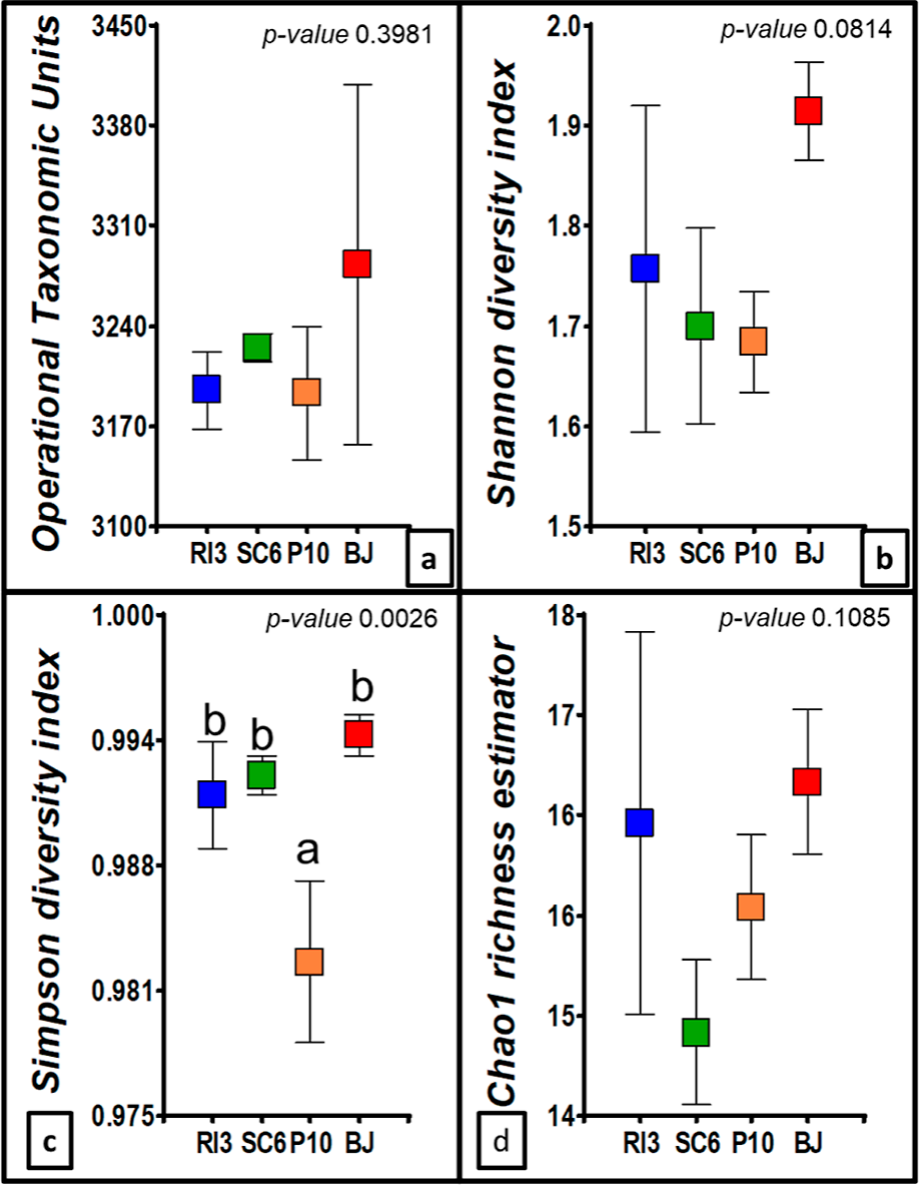
Soil microbial diversity and richness index in the peanut rhizosphere at the R5 phenological stage. (a) OTUs, (b) Shannon diversity index, (c) Simpson diversity index, and (d) Chao1 richness estimator. Legend of treatments apply in all the figures RI3, *B. velezensis*; SC6, *B. velezensis*; P10, *P. psychrophila*; BJ, *B. japonicum*. Error bars indicate standard deviations. Different letters indicate statistical differences (*p*-value < 0.05).

After analyzing the bioinformatic results, we found that the number of OTUs ranged from 3191 to 3283 (Figure 4). No differences were detected between treatments at the OTUs level, indicating a close relation between them. Considering the number and abundance of species, we found that the treatment values for the Shannon diversity index ranged between 1.68–1.91. Shannon diversity index was 12% higher in BJ than the PGPR (RI3 > SC6 > P10). Additionally, the Simpson diversity index was statistically significant, suggesting changes in the diversity of the most abundant species. P10 exhibited the lowest Simpson diversity index value, 1.1% below BJ and 0.8% below RI3 and SC6. Lastly, the Chao1 species richness estimator ranged from 15 to 17 (Figure 4).

### 3.4 Phyla Analysis

**Figure 5:**
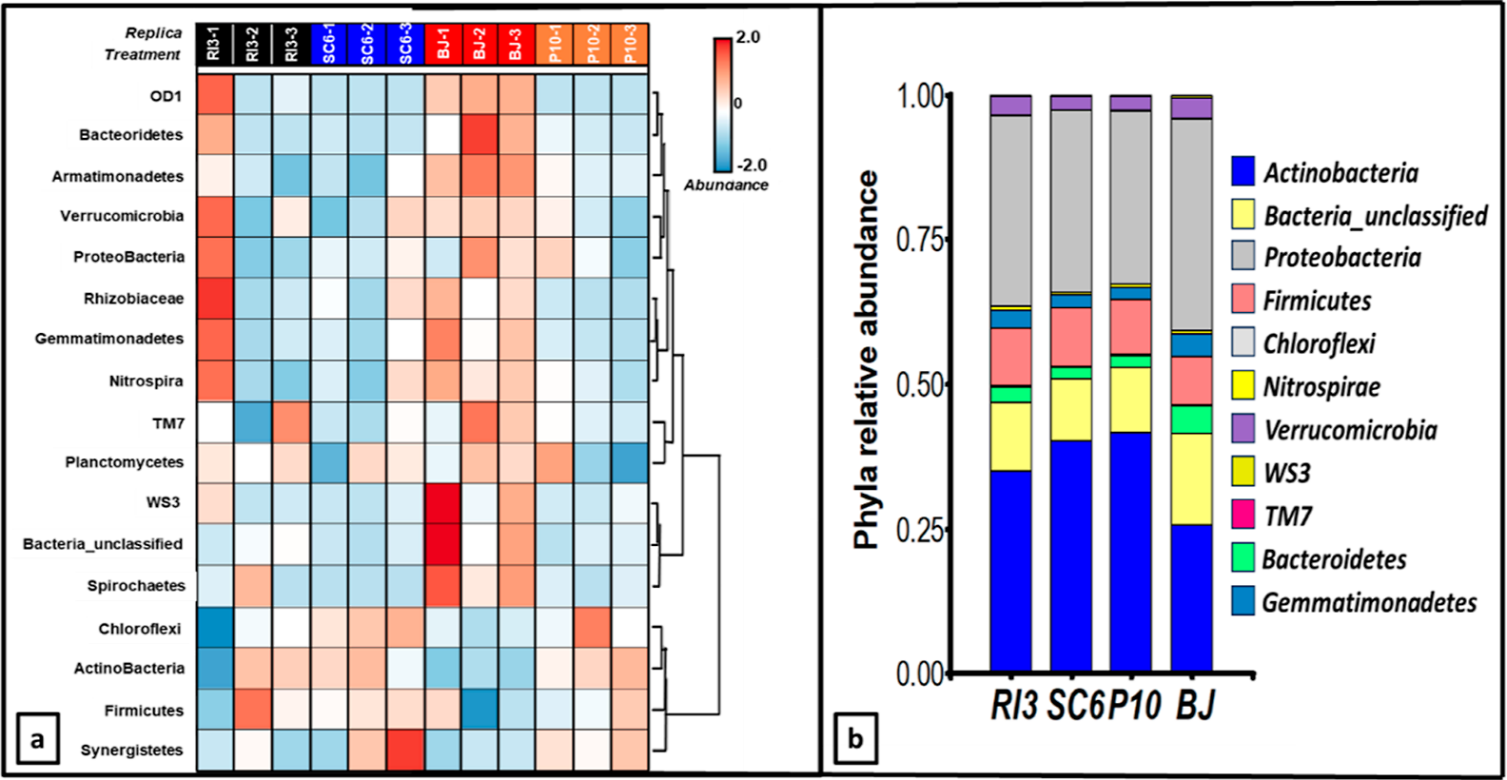
Peanut rhizosphere analysis at the phylum level. (a) Heatmap of phylum composition and abundance associated with peanut rhizosphere at R5 phenological stage. Rows represent different phyla and columns each treatment replicates. The relative abundance of each phylum was depicted by color intensity according to the top-right legend. The varying color codes (Z-scored) indicate the abundance of each phylum, expressed as a value between −2 (low abundance) and 2 (high abundance). (b) Phyla relative abundance. The figure shows the most abundant phyla (Actinobacteria; Proteobacteria; Bacteria_unclassified; Firmicutes; Bacteroidetes; Gemmatimonadales; Verrucomicrobia; Nitrospirae; WS3; Chloroflexi; TM7). Legend of treatments corresponds to RI3, *B. velezensis*; SC6, *B. velezensis*; P10, *P. psychrophila*; BJ, *B. japonicum*.

The phylogenetic analysis of bacteria phyla associated with the peanut rhizosphera could be divided into two major clusters (Figure 5a). Group 1 included several phyla such as Parcubacterias (OD1), Bacteroidetes, Armatimonadetes, Verrucomicrobia, Proteobacteria, Gemmatimonadetes, Nitrospira, Patescibacteria (TM7), Planctomyces, Latescibacteriota (WS3), Bacteria_Unclassified (hereafter “others”), and Spirochaetes phyla. Group 2 consisted of Chloroflexi, Actinobacteria, Firmicutes, and Synergistetes phyla (Figure 5a). Compared to the PGPR treatments, the BJ treatment highly modified the abundance by nearly 2× of those phyla clustered in group 1 (Figure 5a). PGPR, in contrast, increased the relative abundance of species included in group 2, nearly doubling their presence (Figure 5a). The results of major phyla abundance are presented in Figure 5a. PGPR exhibited 20% to 88% lower abundance in the phyla belonging to group 1 compared to BJ. In contrast, the abundance of the phyla in group 2 under the influence of PGPR was 17% to 62% higher than BJ (Figure 5a).

Considering the most abundant phyla, there were differences in their relative abundance between treatments in Actinobacteria, Proteobacteria, Firmicutes, Bacteroidetes, Gemmatimonadetes, Verrucomicrobia, Nitrospirae, WS3, and Others (Figure 5b). Actinobacteria and Proteobacteria were the most abundant phyla, accounting for 70% of the phyla identified in the PGPR treatments. For Actinobacteria, RI3 (0.44) and P10 (0.42) treatments exhibited higher proportions than BJ (0.26). In the case of Proteobacteria, at the PGPR level, SC6 (0.31) showed higher levels of Proteobacteria compared to RI3 and P10. Nevertheless, BJ showed the highest level of Proteobacteria (0.36). Considering the three most abundant phyla (Actinobacteria > Proteobacteria > Firmicutes), P10 treatment showed 82% of the microorganisms belonging to these phyla, followed by SC6 at 81%, RI3 at 80%, and BJ at 70% (Figure 5b).

### 3.5 Genera Analysis

**Figure 6:**
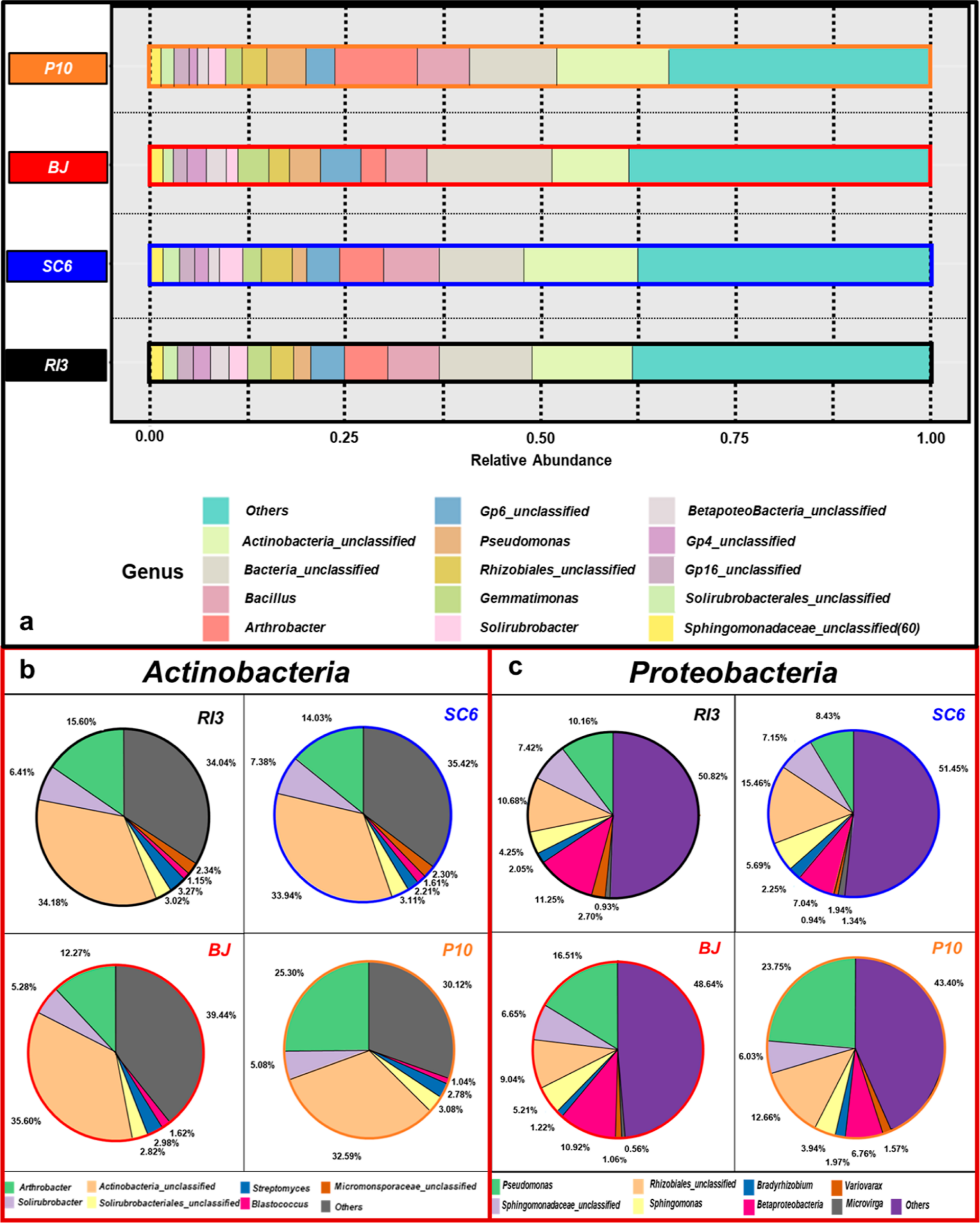
Genera analysis of the peanut rhizosphere at the R5 phenological stage. (a) Taxonomic composition of peanut rhizosphere at R5 phenological stage and genus level without considering the phylum belonging. The figure (a) included genera whose relative abundance was greater than 0.005. Rows bars represent different treatments. (b) Genera relative abundance in Actinobacteria phylum. (c) Genera relative abundance in Proteobacteria phylum. (b,c) Both phyla represented, on average, 49–62% (BJ < RI3 < SC6 < P10) of the total microorganisms identified in the peanut rhizosphere. The treatment legend of the charts corresponds to RI3, *B. velezensis*; SC6, *B. velezensis*; P10, *P. psychrophila*; BJ, *B. japonicum*. The legend at the bottom of each figure indicates by color each genus.

The peanut rhizosphere showed modifications at the genera level (Figure 6), with RI3 and SC6 (both *B. velezensis*) exhibiting similar abundance patterns (Figure 6a). The analysis of genera abundance across all treatments revealed that more than 35% fell into the “other” category, with over 40% classified as “unclassified” (Figure 6a; Table S1). Arthrobacter, Pseudomonas, and Bacillus were the most prevalent and accurately identified genera across the treatments. RI3 and SC6 showed the highest levels of *Bacillus* genera, ranging from 5% to 8% (Figure 6a; Table S1). P10 showed higher abundances for *Arthrobacter* (6%) and *Pseudomonas* (11%) genera, compared to RI3 and SC6, which exhibited 7% (*Arthrobacter*) and 1.5% (*Pseudomonas*) (Figure 6a; Table S1).

Considering that the most abundant genera (*Arthrobacter*, *Pseudomonas*, and *Bacillus*) belong to the most abundant phyla, Actinobacteria, Proteobacteria, and Firmicutes, we analyzed the changes in the genera composition of each phylum (Figures 6b,c and S1). Regarding Actinobacteria phylum (Figure 6b), Actinobacteria_unclassified showed the highest values for BJ (35.60%) > RI3 (34.18%) > SC6 (33.94%) > P10 (32.59%). P10 exhibited the highest abundance for *Arthrobacter* (25.3%), which was 81% higher than RI3 > SC6 > BJ. *Solirubrobacter* was more abundant in SC6 (7.39%), 40% above BJ, and 45%–15% higher than in P10 and RI3 (Figure 6a). *Streptomyces* showed higher abundance in RI3 (3.27%) than BJ (2.98%). On the other hand, *Blastococcus* was higher in SC6 (1.61%) than in P10 (1.04%) (Figure 6b).

Regarding Proteobacteria phylum (Figure 6c), Pseudomonas, Rhizobiales_unclassified, and Betaproteobacteria accounted for 36% on average. P10 (P. psychrophila), which belongs to the *Pseudomonas* genera, exhibited from 43% to 181% higher abundance than BJ > RI3 > SC6. In the case of Rhizobiales_unclassified, the PGPRs (SC6 > P10 > RI3) were 71%–18% higher than BJ. Considering Betaproteobacteria, RI3 exhibited the highest abundance, 3% more than BJ and over 60% higher than P10 and SC6. The same trend was observed in Sphingomonadacea_unclassified and *Sphingomonas* at P10, which were between 15% and 22% lower than RI3 > BJ > SC6. *Bradyrhizobium* genera abundance was 1.22% at BJ treatment (*B. japonicum*), 42% lower than the other treatments SC6 > RI3 > P10 (2.25%–1.97%) (Figure 6c).

Additionally, differences were found in the genera distribution in the Firmicutes phylum, considering that RI3 and SC6 were Bacillus strains (Figure S1). In both strains, >78% of the identified microorganisms correspond to the Bacillus genus. SC6 exhibited 12% higher microorganisms for the Bacillus genus than BJ. In the case of RI3, it was also higher than BJ (6%). Considering P10, it was 8% higher than BJ and 5% lower than SC6.

### 3.6 Functional Gene Analysis

**Figure 7:**
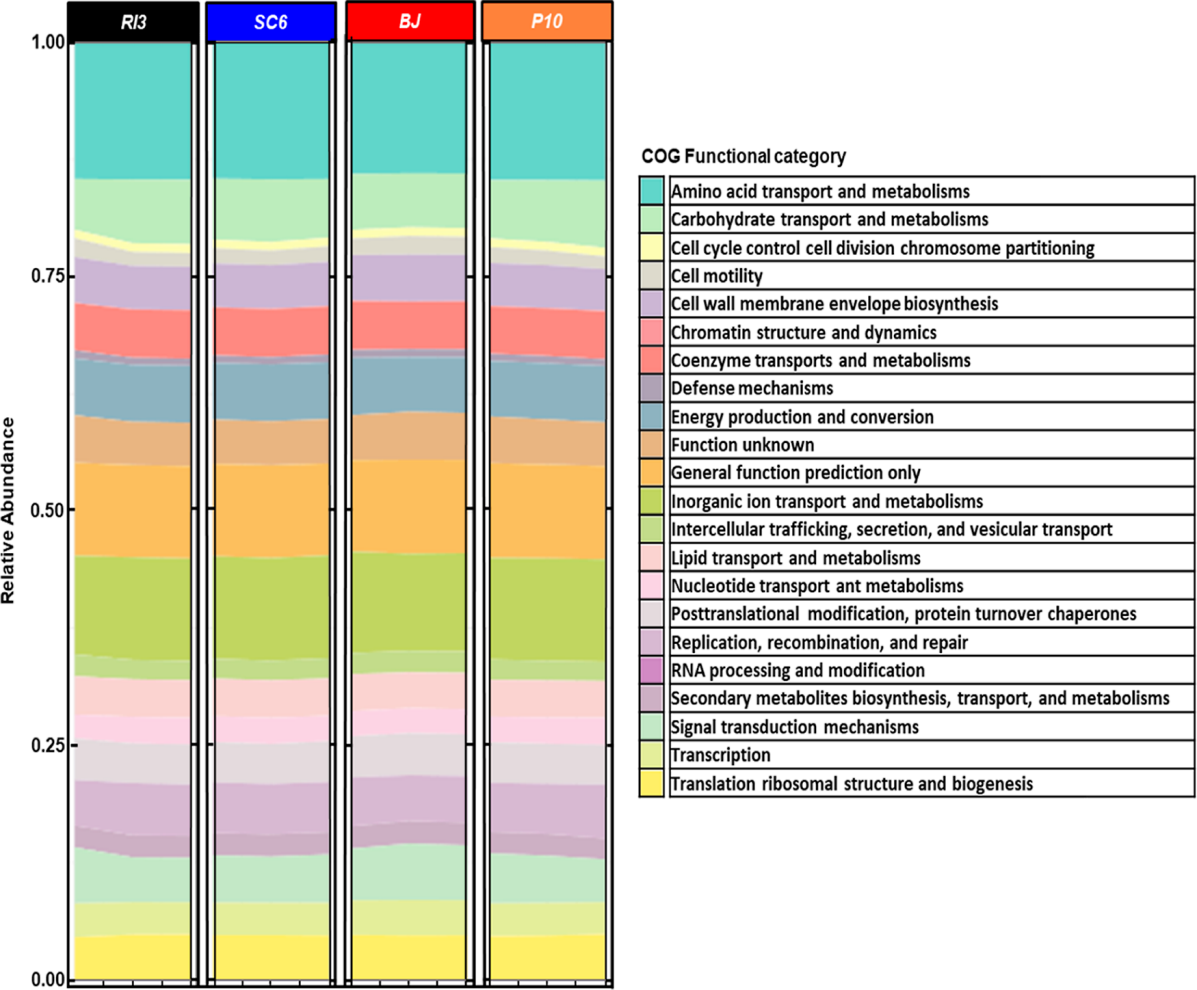
Gene functional analysis based on the taxonomic composition of peanut rhizosphere at R5 phenological stage. Columns represent different treatments, and the legend corresponds to RI3, *B. velezensis*; SC6, *B. velezensis*; P10, *P. psychrophila*; BJ, *B. japonicum*. Each stacked bar plot corresponds to three replicates and displays the metabolic pathway abundance for each process. Legend (color) in the figures corresponds to each metabolic process predicted based on the COG (clusters of orthologous groups) functional categories system, which is used to classify genes based on their predicted function.

Other information obtained from the rhizosphere metagenome analysis were bacterial metabolic pathways (Figure 7). The metabolic processes predicted throughout the COG functional categories system revealed different metabolic pathways of the metabolism categorized into different groups, including the metabolism of amino acids, carbohydrates, energy production, inorganic ions, lipids, nucleotides, and secondary metabolites, as well as cellular processes, genetic information processing (GIP), and environmental information processing (EIP) (Figure 7). In terms of the general metabolism process (GMP), PGPR (P10 > SC6 > RI3) showed the highest relative abundance from 52.06% to 52.40% compared to BJ (50.44%) (Figure 7; Table S2). The transport and metabolism of amino acids and carbohydrates represent the major GMP in the rhizosphere, and the PGPR was between 5% and 10% above BJ (Figure 7; Table S2). Considering GIP and EIP, BJ achieved the highest relative abundance, 24.79%, and 3.15%, respectively (Figure 7; Tables S3 and S4). In GIP, the posttranslational modification protein turnover chaperones, signal transduction mechanisms, and transcription mechanisms were reduced in the PGPR treatments by 3% to 13% compared to BJ (Table S4). On the other hand, on average, DNA replication, recombination, and repair were increased by 7% (Table S4). Cellular processing was reduced in the PGPR treatments by 1% to 14% concerning BJ (Table S5).

Considering the significance of Nitrogen for legumes, the gene analyses showed that PGPRs modified the relative abundance of OTUs mapped to selected N-cycle genes (Table 3). Generally, PGPRs decreased the abundance of genes related to biological nitrogen fixation and nitrification processes concerning the noninoculated control (BJ). Regarding biological nitrogen fixation, the reduction in abundance ranged from 8% to 14%, with RI3 being the exception, showing a 15% increase in the genes *nifD* and *nifK*. In the case of nitrification-related genes, the reduction was significant, ranging from 80% to 10% (P10 > SC6 > RI3). Considering the nitrogen assimilation process (*nasA* and *nirA*), PGPRs increased the abundance of these genes by 28% to 160% (RI3 > P10 > SC6). Genes related to denitrification showed increased abundance, such as *nirK*, by 400%, and decreased genes, such as *nisZ*, by 14% and *norB*, by 6% relative to BJ. The gene related to glutamine synthetase (*gln A*) also decreased by 33% (Table 3).

**Table 3:** Effect of PGPR on Relative Abundance (%) of OTUs Mapped to Selected N Cycle Genes (*n* = 3)

treatments	*nirA*	*nirK*	*narG*	*nasA*	*nosZ*	*ureC*	*gln A*	*nifD*	*nifH*	*nifK*	*norB*	*hao*	*amoA*
RI3^a^	0.84 a^b^	19.2 b	3.29	0.72 a	0.44 b	5.23	0.15 c	5.17 a	7.09 b	5.17 a	5.82 c	0.51 b	7.33 a
SC6	0.15 c	25.7 a	3.72	0.31 b	0.85 a	4.92	0.46 b	3.77 bc	6.96 b	3.78 bc	7.62 a	0.49 b	5.14 b
P10	0.59 b	15.4 c	3.46	0.75 a	0.15 c	5.10	0.85 a	3.43 c	5.65 c	3.42 c	5.28 c	0.15 c	2.45 c
BJ	0.41 b	3.3 d	3.25	0.23 b	0.56 b	5.07	0.54 b	4.48 ab	7.6 a	4.48 ab	6.61 b	0.85 a	8.14 a
*PGPR*^c^	<0.001	<0.001	NS	0.005	0.001	NS	<0.001	0.005	<0.001	0.005	<0.001	<0.001	<0.001

a LS means with different letters are significantly different (LSD Fisher posthoc test, *p* < 0.05).

b RI3, *B. velezensis*; SC6, *B. velezensis*; P10, *P. psychrophila*; BJ, *B. japonicum*.

c *p*-values; NS, no significant.

## 4 Discussion

This study addressed the effects of PGPR on peanut yield generation under field conditions by examining changes in microbial activity and soil microbiome structure. Our research highlights the positive impact of PGPR on peanut yield and soil microbial dynamics at the crop level, an aspect that previous studies at the plant scale have overlooked.^13,20,21,23^ This study showed that field applications of PGPR in rainfed conditions increased the SY by 8%. Bigatton et al.^10^ observed similar behavior using the same strains under irrigated peanuts. The increase in SY was due to a 10% rise in RUE, similar to other research findings that reported a 12–18% increase in RUE.^10,31^ The improved RUE led to a 13% increase in TB, resulting in higher assimilate partition rates and an increase in PN (9.5%) and SN (8.5%), major yield components and sinks that strengthen canopy activity.^2,10,31−34^ Also, PGPR positively modified soil enzymes (FDA 17% and DHA 28%) and increased microbial abundance and activity during the SY definition stage (R5). Microbial diversity indices did not show significant differences; nonetheless, changes in the most abundant phyla and genus were observed (Simpson index). Several studies suggest that PGPR applications could modify the rhizosphere microbial composition directly or indirectly through changes in the plant exudates and dynamics.^35^ In this study, the PGPR-peanut rhizosphere increased the relative abundance of Proteobacteria and Actinobacteria phyla, accounting for 70% of the microorganisms detected. This outcome aligns with modifying the peanut rhizosphere through stresses or biological amendments.^21,36^ Considering the genus analysis, Bacillus and Pseudomonas, novel PGPR genera, were some of the dominant genera detected in the PGPR-peanut rhizosphere and novel PGPR strains.^17,20,37^

The peanut SY significantly improved throughout the PGPR application (SC6 > RI3 > P10) from 11–3%. Several studies have demonstrated that PGPR can increase crop yield by 5–50%. The effects of growth promotion depend not only on the microorganism itself but also on interactions between genotypes, environments, nutrient availability, and, as in the case of this study, interactions with the soil microbial community.^31,32,34^ A positive relationship between SY and SN has been proven in peanut cultivation.^2^ In our study, SN was highly correlated to SY (*R*^2^ > 0.90), and these yield component modifications are crucial for understanding the yield generation dynamics by PGPR. Yield component increases are primarily promoted by changes in flowering patterns that positively impact the generation of reproductive structures (e.g., PN).^38−41^ A greater PN is associated with a higher SN and seeds per pod.^2^ We also observed the SW and SF > 8 mm changes, which impact the SY determination and peanut commercial value, respectively. Another explanation for the SY improvement was that the PGPR increased TB (7–19%). Since there is a linear relationship between SY and TB (i.e., harvest index), the higher TB with a harvest index value close to 0.29 (data not shown), consistent with the literature, implies a higher SY.^2,10^ From an efficiency viewpoint, the observed increase in SY might be attributed to RUE improvement (7–14%; 1.90–2.03 g MJ^–1^). These values are consistent with the literature for peanut cultivation (1.89–2.30 g MJ^–1^),^2,10^ and the increments align with findings from other PGPR studies.^31^ The RUE improvement may be explained by a strengthened sink of assimilates (i.e., SN), leading to increased source (canopy) activity and, subsequently, higher photosynthetic rates.^2,42^ The extensive root proliferation in peanuts induced by PGPR (46–88%) has led to improved exploration of the soil profile.^43^ The correlations between crop performance and rhizospheric activity may elucidate the efficiency and resource uptake improvements.^15^

Soil rhizospheric activity at the R5 peanut phenological stage was significantly enhanced throughout the PGPR treatment. Research indicates that soil rhizospheric population and biological activity reach their maximum at the crop yield determination stage.^44^ Our study determined that the PGPR application increased the FDA and DHA by an overall 20%. In sunflower^45^ and maize,^46^ similar increases in the FDA and DHA under bioformulation application (ca. + 20%) have been demonstrated. Enzyme enhancements indicate soil quality and health in the PGPR-plant interactions.^45−47^ PLFA analyses provided information about changes in soil microbial structure and were consistent with other soil regional research.^29,48^ Upon conducting a comparative analysis between PGPR and BJ, the results were consistent with Chaudhary et al.,^49^ who treated peanuts with PGPR and showed similar significant changes in the PLFA analysis for GP, GN, F, ACT, and AMF (ca. + 30%). Soil rhizospheric activity determines soil fertility, structure, and overall ecosystem function.^50^ PGPR treatments interact with plant and native microbiomes through specific relationships, determining plant growth and health.^15,16^

The findings obtained in the PLFA analysis aligned with the metagenomic approach. PLFA highlighted significant changes in the soil microbial structure in terms of abundance, as evidenced by the metagenomics analysis. Metagenomics analysis through the Shannon index indicates that applying PGPR treatments did not significantly alter the species richness and diversity at the community level in the peanut rhizosphere. These findings suggest that using PGPR treatments may have a minimal impact on the ecological composition of the soil microbiome.^15,49,51,52^ However, the PGPR application modified the relative abundance of the two major phyla detected (∼70%) in the peanut rhizosphere, Actinobacteria (62%) and Proteobacteria (−20%).^20−22^ These phyla were the most abundant in the plant rhizosphere because they are copiotrophs and play roles like nutrient cycling and solubilization (e.g., P and K), N fixing (free-living, symbiotic, or diazotrophic bacteria), plant hormone production, and many antistress compounds (biotic and abiotic stresses).^53,54^ Firmicutes, Bacteroidetes, and Gemmatinomonas phyla (∼14% of the total phyla detected) also exhibited changes and are important because they promote nutrient and carbon cycling and plant growth.^53,54^ Peanut rhizospheric microbiome at the phyla level demonstrated that PGPR could favor plant-growth beneficial phyla (higher relative abundance).^20,36^ Furthermore, these results demonstrate that plants determine the structure of their microbiome and the persistence over time of PGPR effects.^55^

PGPR effects on the peanut-field rhizosphere could be explained at the microbiome genera level. Several studies demonstrated that genera belonging to Actinobacteria, Proteobacteria, and Firmicutes, such as *Arthrobacter*, *Streptomyces*, *Pseudomonas*, *Rhizobium*, *Bradyrhizobium*, *Sphingomonas*, *Bacillus*, *Paenibacillus*, among others, were increased during the crop yield definition stage.^19−21^*Bacillus*, *Arthrobacter*, and *Pseudomonas* were the three most abundant genera identified in the peanut rhizosphere. These genera are widely known as PGPR, and their association with the crops enhances yield and allows for the overpassing of stresses (biotic and abiotic).^56^ RI3 and SC6 (both *B. velezensis*) showed the highest values of Bacillus abundance (8%), similar to other reports that used Bacillus as PGPR in maize^17^ and peanuts.^20^ Considering Pseudomonas genera, P10 (*P. psychrophila*) exhibited a higher relative abundance (6%), such as other studies in maize^37^ and peanuts.^57^ Peanut crops establish symbiotic relationships with multiple species within the Bradyrhizobium genus.^6−8^ Several studies have established a positive correlation between using BJ (*B. japonicum*) and enhanced crop yield.^6,8,10^ Metagenomic analysis at the genus level indicated that PGPR treatments increased the relative abundance of Bradyrhizobium (1.97–2.25%) compared to BJ (1.22%) treatment. These findings align with previous studies that have demonstrated the positive impact of PGPR on rhizobia populations.^20,21,57^ Metagenomic analysis also described the bacterial metabolic pathways throughout the COG functional categories system. These results revealed important changes related to the general metabolic process and could be correlated with an increased abundance of copiotrophic microorganisms enhanced by the PGPR. These results showed that PGPRs were functionally active, inducing crop growth and yield generation at the crop level. Additionally, considering the significance of N in the legume crops, the gene analysis demonstrated that PGPRs modified the relative abundance of N-cycle genes and consequently the N metabolisms and rhizosphere dynamic.^58,59^ PGPRs decrease the relative abundance of genes associated with N-fixation and nitrification while increasing genes related to N-assimilation and N availability in the rhizosphere.^59,60^ For instance, the increased relative abundance of the *nirK* gene in PGPRs suggests its potential role in nitric oxide (NO) production and its impact on plant growth and rhizosphere signaling.^60,61^ These findings highlight the need for further research to validate these results in different crop scenarios, considering that most studies have been conducted in controlled pot environments.

Overall, the present study proved that applying PGPR positively impacts peanut yield and soil microbiome structure. Under rainfed conditions, PGPR increased SY, attributed to enhancements in SY components (SN) and improvements in the RUE. In addition, PGPR positively modified soil enzymes and increased microbial abundance and activity during the SY definition stage (R5). Our research highlights microbiome parameters that indicate improved soil health. While PGPR did not significantly alter microbial diversity, it did affect the relative abundance of key phyla (Actinobacteria > Proteobacteria > Firmicutes) and genera (*Bacillus* > *Arthrobacter* > *Pseudomonas*), particularly favoring copiotrophic microorganisms beneficial for plant growth. These findings highlight the potential of PGPR as a sustainable agricultural practice to enhance peanut production and soil fertility.

## Data Availability

The 16S rRNA gene sequences were deposited ENA/NCBI (PROJECTPRJEB74143).

## Supporting Information Available

Genera distribution in Firmicutes phylum at peanut rhizosphere at R5 phenological stage. Relative taxonomic distribution at the genera level without considering the phyla belonging. Metabolic pathways include general metabolic processes, cellular processes, genetic information processes, and environmental information processes in function of the taxonomic composition of peanut rhizosphere at R5 (PDF)
