# Supplementary material for: Metagenomic Analysis to Assess the Impact of Plant Growth-Promoting Rhizobacteria on Peanut (Arachis hypogaea L.) Crop Production and Soil Enzymes and Microbial Diversity
Source: J Agric Food Chem. 2024 Sep 26;72(40):22385–97. doi: 10.1021/acs.jafc.4c05687 (PMC11468012; doi:10.1021/acs.jafc.4c05687)
Supplement: Supplementary file 1 — jf4c05687_si_001.pdf [file jf4c05687_si_001.pdf]

Metagenomic analysis to assess the impact of plant growth-promoting rhizobacteria on peanut (*Arachis hypogaea* L.) crop production and soil enzymes and microbial diversity

Ezequiel D. Bigatton[a,b,c*], Romina A. Verdenelli[d], Ricardo J. Haro[e], Ibrahim Ayoub[a,b], Florencia M. Barbero[d], Maria Paula Martín[a], Lucas E. Dubini[a], Jesús V. Jorrín Novo[c], Enrique I. Lucini[a], María Ángeles Castillejo[c*].

[a] Universidad Nacional de Córdoba, Facultad de Ciencias Agropecuarias, Microbiología Agrícola, Ingeniero Agrónomo Félix Aldo Marrone 746, Córdoba, Córdoba, X5000, Argentina.

[b] Consejo Nacional de Investigaciones Científicas y Técnicas (CONICET), Av. Ciudad de Valparaíso S/N, Córdoba, Córdoba, X5016, Argentina.

[c] Universidad de Córdoba, Departamento de Bioquímica y Biología Molecular-ETSIAM, AGR-164 Bioquímica, Proteómica y Biología de Sistemas Vegetal y Agroforestal, Autovía N-IV Km 396, Campus Rabanales, Córdoba, Andalucía, 14071, España.

[d] Instituto Multidisciplinario de Biología Vegetal (IMBIV-CONICET-UNC), Instituto de Ciencia y Tecnología de los Alimentos (FCEFyN-UNC), Av. Vélez Sarsfield 1666, Córdoba, Córdoba, X5016, Argentina.

[e] Instituto Nacional de Tecnología Agropecuaria (INTA), Estación Experimental Agropecuaria INTA Manfredi, Ruta Nacional Nº9 Km 636, Manfredi, Córdoba, X5988, Argentina.

*Email: ezequiel.bigatton@unc.edu.ar or z82bibie@uco.es
*Email: bb2casam@uco.es

Supplementary Materials

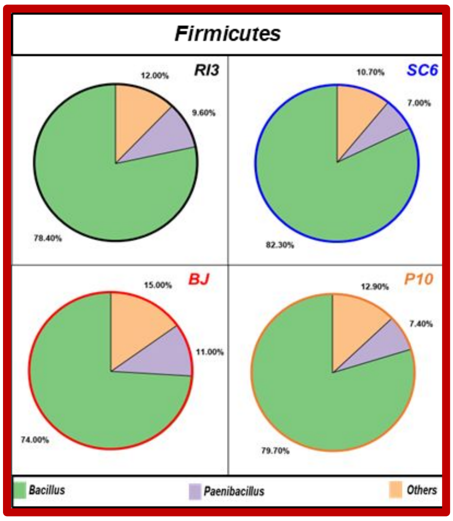

Figure S1. Genera distribution of Firmicutes in the peanut rhizosphere at R5. The legend at the top of the charts corresponds to **RI3**, *B. velezensis*; **SC6**, *B. velezensis*; **P10**, *P. psychrophila*; **BJ**, *B. japonicum*. The legend at the bottom indicates by color each genus.

Table S1. Relative taxonomic composition of peanut rhizosphere at and genera level at R5 phenological stage. The table shows the most abundant genus in column (Actinobacteria_unclassified; Bacteria_unclassified; *Bacillus*; *Arthrobacter*; Gp6; *Pseudomonas*; Rhizobiales_unclassified; *Gemmatimonas*; *Solirubrobacter*; Betaproteobacteria_unclassified; Gp4; Gp16; Solirubrobacterales_unclassified; Sphingomonas_unclassified).

PGPR	Actinobacter_ unclassified	Bacteria_ unclassified	*Bacillus*	*Arthrobacter*	Gp6	*Pseudomonas*	Rhizobiales _unclassified	*Gemmatimonas*	*Solirubrobacter*	B
RI3*	0.16	0.12	0.08	0.07	0.03	0.01	0.03	0.02	0.03 a	0
SC6	0.15	0.11	0.07	0.06	0.02	0.02	0.04	0.02	0.03 a	0
P10	0.15	0.11	0.07	0.11	0.01	0.06	0.03	0.02	0.02 ab	0
BJ	0.10	0.16	0.05	0.03	0.03	0.05	0.03	0.04	0.01 b	0

* **RI3**, *B. velezensis*; **SC6**, *B. velezensis*; **P10**, *P. psychrophila*; **BJ**, *B. japonicum*.

Table S2. Abundance (%) of metabolic pathways in the function of the taxonomic composition of peanut rhizosphere at R5 phenological stage. The table shows the most abundant general metabolic processes in column (amino acid transport and metabolisms; carbohydrate transport and metabolisms; energy production and conversion; inorganic ion transport and metabolisms; lipid transport and metabolisms; nucleotide transport and metabolisms; and secondary metabolites biosynthesis, transport, and metabolisms).

**PGPR**	**Amino acid transport and metabolisms**	**Carbohydrates transport and metabolisms**	**Coenzyme transport and metabolisms**	**Energy production and conversion**	**Inorganic ion transport and metabolisms**	**Lipid transport and metabolisms**	**Nucleotide transport and metabolisms**	**Secondary metabolites biosynthesis, transport, and metabolisms**
RI3*	14.59	6.38	5.11	6.07	10.80	4.04	2.73	0.03
SC6	14.56	6.48	5.13	6.06	10.96	4.00	2.74	0.04
P10	14.69	6.66	5.12	5.97	10.57	3.92	2.82	0.03
BJ	13.97	5.86	5.09	5.95	10.92	3.86	2.68	0.03

* **RI3**, *B. velezensis*; **SC6**, *B. velezensis*; **P10**, *P. psychrophila*; **BJ**, *B. japonicum*.

Table S3. Abundance (%) of metabolic pathways in the function of the taxonomic composition of peanut rhizosphere at R5 phenological stage. The table shows the most abundant cellular processes in the column (cell motility, cell wall membrane, general function).

**PGPR**	**Cell motility**	**Cell wall membrane envelope biosynthesis**	**General function**

RI3*	1.65	6.38	9.88
SC6	1.61	6.48	9.86
P10	1.53	6.66	10.02
BJ	1.88	5.86	9.22

* **RI3**, *B. velezensis*; **SC6**, *B. velezensis*; **P10**, *P. psychrophila*; **BJ**, *B. japonicum*.

Table S4. Abundance (%) of metabolic pathways in the function of the taxonomic composition of peanut rhizosphere at R5 phenological stage. The table shows the most abundant genetic information process in column (cell cycle control, cell division, and chromosome partitioning; chromatin structure and dynamics; posttranslational modification, protein turnover chaperones; DNA replication, recombination, and repair; RNA processing and modification; signal transduction mechanism; transcription; translational ribosomal structure and biogenesis).

PGPR	Cell cycle control, cell division, and chromosome partitioning	Chromatin structure and dynamics	Posttranslational modification, protein turnover chaperones	DNA replication, recombination, and repair	RNA processing and modification	Signal transduction mechanism	Transcription	Translational ribosomal structure and biogenesis
RI3*	0.92	0.02	4.29	5.28	0.03	5.17	3.49	4.75
SC6	0.92	0.02	4.29	5.36	0.03	5.04	3.46	4.77
P10	0.92	0.02	4.25	5.42	0.03	4.98	3.45	4.78
BJ	0.93	0.02	4.43	4.98	0.03	5.78	3.73	4.77

* **RI3**, *B. velezensis*; **SC6**, *B. velezensis*; **P10**, *P. psychrophila*; **BJ**, *B. japonicum*.

Table S5. Abundance (%) of metabolic pathways in the function of the taxonomic composition of peanut rhizosphere at R5 phenological stage. The table shows the most abundant environmental information process in the column (defense mechanisms, intercellular trafficking secretion, and vesicular transport).

PGPR	Defense mechanisms	Intercellular trafficking secretion and vesicular transport
RI3*	0.81	2.16
SC6	0.80	2.13
P10	0.76	2.10
BJ	0.88	2.27

* **RI3**, *B. velezensis*; **SC6**, *B. velezensis*; **P10**, *P. psychrophila*; **BJ**, *B. japonicum*.
